# Successive Self-Nucleation and Annealing for the Characterization of Biomedical Ultra-High-Molecular-Weight PolyEthylene (UHMWPE) Formulations

**DOI:** 10.3390/polym18121428

**Published:** 2026-06-08

**Authors:** Luca Gianoglio, Matteo Righetti, Marco Zanetti, Pierangiola Bracco

**Affiliations:** 1 Department of Chemistry, University of Torino, Via Pietro Giuria 7, 10125 Turin, Italy; luca.gianoglio@unito.it (L.G.); matteo.righetti@unito.it (M.R.); marco.zanetti@unito.it (M.Z.); 2 SUSPLAS@UniTo, Sustainable Plastic Scientific Hub, University of Torino, Via Pietro Giuria 7, 10125 Turin, Italy; 3 NIS Interdepartmental Centre, University of Torino, 10125 Turin, Italy

**Keywords:** UHMWPE, successive self-nucleation and annealing, DSC, thermal fractionation

## Abstract

The Successive Self-Nucleation and Annealing (SSA) technique is a thermal fractionation method that involves subjecting a polymer sample to sequential self-nucleation and annealing steps at progressively decreasing temperatures, using differential scanning calorimetry (DSC). Since its introduction in the late 1990s, SSA has been widely applied to study the molecular structure of polymers with structural irregularities, including highly branched or crosslinked polyethylenes and random copolymers. However, the use of SSA for medical-grade ultra-high-molecular-weight polyethylene (UHMWPE), a highly linear homopolymer with minimal defects, has not yet been explored. This study aims to evaluate both its applicability to biomedical UHMWPE and its ability to reveal morphological differences among commercially available formulations. Several biomedical UHMWPE formulations, including conventional, highly cross-linked, and α-tocopherol-stabilized materials, were characterized by micro-FTIR, gel fraction and cross-link density measurements and subsequently subjected to SSA thermal fractionation. The results show that ram extrusion induces entanglements that act as interruptions in the otherwise linear chain structure, thereby enabling thermal fractionation: more than 80% of the crystalline fraction of ram-extruded UHMWPE is composed of three crystal populations melting at approximately 135, 132, and 126 °C, accompanied by four additional minor fractions at progressively lower melting temperatures. Gamma irradiation followed by thermal treatments significantly modifies the fractionation behavior, leading to the formation of an additional population of high-melting crystallites as evidenced by an increase in the number of melting peaks from 7 to 8. Oxidative degradation of highly crosslinked and annealed UHMWPE increases crystallinity by approximately 11% relative to its unoxidized counterpart but reduces the ability of the material to undergo thermal fractionation, decreasing the number of melting peaks. In contrast, the addition of low concentrations of α-tocopherol does not significantly influence the fractionation behavior. These findings demonstrate that thermal fractionation of medical-grade UHMWPE is feasible and that SSA is an effective tool for detecting morphological differences among formulations.

## 1. Introduction

### 1.1. Biomedical UHMWPE

Ultra-high-molecular-weight polyethylene (UHMWPE) is a linear, semi-crystalline homopolymer characterized by an extremely high molecular weight and has been used as a bearing material in total joint arthroplasty for more than five decades [1]. It typically exhibits a molecular weight ranging from 3.5 to 6 million g/mol and a degree of crystallinity of approximately 50–55%. The molecular structure of UHMWPE consists of two different phases: a crystalline phase composed of ordered lamellae, in which the chains are arranged in an orthorhombic lattice, and an amorphous phase, where the chains are highly entangled and act as tie molecules between adjacent lamellae [2,3]. These two phases synergistically determine the material properties: the crystalline phase mainly influences modulus and stiffness, whereas the amorphous phase contributes to ductility and toughness.

The first use of UHMWPE in orthopedic applications dates back to 1962. This material, commonly referred to as ‘historical polyethylene,’ has a well-documented clinical history until the late 1990s, and is composed of virgin UHMWPE sterilized with 25–40 kGy of gamma radiation in air [1]. However, it was later recognized that gamma sterilization in air initiated oxidative degradation processes directly associated with osteolysis phenomena, due to the extensive generation of UHMWPE wear particles [4,5]. This finding led to a shift toward irradiation with γ-rays in a low-oxygen environment, such as under vacuum or with inert gases, like argon or nitrogen, which significantly reduces oxidation [1].

On the other hand, irradiation-induced scission of C–C bonds in UHMWPE chains generates free radicals which, under low-oxygen conditions, predominantly lead to crosslinking, thereby improving wear resistance [6,7]. With the aim of maximizing the wear performance of the material, manufacturers developed Highly Cross-Linked Polyethylene (HXLPE) during the 1990s and 2000s. The conventional manufacturing route involves irradiation with a high dose of gamma radiation (typically around 100 kGy) to induce crosslinking, followed by a thermal treatment aimed at eliminating residual free radicals and thus preventing subsequent oxidative degradation.

Two main post-irradiation thermal treatments have been developed over the years, commonly referred to as “annealing” and “remelting.” Annealing consists of a thermal treatment performed below the polymer melting temperature (typically 110–120 °C), whereas remelting involves heating above the melting temperature of the polymer (typically around 150 °C) [8].

However, the annealing treatment leaves most of the crystalline phase unaltered and is therefore unable to promote the recombination of all the free radicals generated during irradiation. As a consequence, a measurable concentration of residual free radicals remains trapped within the polymer matrix after annealing. Over time, these radicals may react with oxygen, thereby initiating the oxidative degradation process [9]. In contrast, remelting effectively promotes the recombination of the irradiation-induced free radicals, resulting in a substantially radical-free material with improved oxidation stability compared to annealed HXLPE [10,11]. However, the remelting process also reduces the crystallinity of the polymer, raising concerns regarding its fatigue resistance.

An alternative strategy for improving oxidation stability involves the incorporation of antioxidant stabilizers into HXLPE formulations. This approach eliminates the need for post-irradiation thermal treatment, thereby preserving the original morphology, mechanical properties, and fatigue resistance of the material [12].

As early as the 1990s, some researchers demonstrated that biocompatible stabilizers could be useful, or even necessary, for improving the oxidative stability of orthopedic UHMWPE [13]. Among them, α-tocopherol (synthetic vitamin E) has become the most widely used natural antioxidant. Its stabilization mechanism has been extensively investigated in polymer processing and has shown superior effectiveness compared to many conventional commercial additives [12,14]. In addition, α-tocopherol is an FDA-approved additive for food and beverages, with an annual commercial production exceeding 50,000 tons. Vitamin E is soluble in UHMWPE, exhibiting a solubility of approximately 3 wt% at room temperature, while remaining insoluble in water and synovial fluid, thus preventing its easy extraction from UHMWPE prosthetic components [15].

Vitamin E can be incorporated into HXLPE through two different approaches: before irradiation and crosslinking (“vitamin E-blended”) or after the crosslinking process (“vitamin E-doped”) [2,16]. The first approach allows a more precise and homogeneous incorporation of the antioxidant within the polymer matrix, although it may partially interfere with the crosslinking reaction. In contrast, post-irradiation doping generally results in a spatially non-uniform distribution of α-tocopherol, requiring an additional thermal treatment to achieve a homogeneous antioxidant concentration throughout the material thickness [17,18].

### 1.2. Successive Self–Nucleation and Annealing Technique

The Successive Self-Nucleation and Annealing (SSA) technique is a thermal fractionation method originally introduced by Müller et al. in 1997 [19]. The method relies on the sequential application of self-nucleation and annealing steps to a polymer sample at progressively decreasing temperatures, using differential scanning calorimetry (DSC). Following this thermal conditioning, the final heating run yields a distribution of melting temperatures, induced by the SSA protocol, reflecting the heterogeneity of the polymer chain structure under investigation.

According to its developers, SSA combines relatively short analysis times with a very high fractionation resolution, compared to other thermal fractionation techniques [20,21].

In principle, effective thermal fractionation requires a certain degree of structural heterogeneity within the polymer chains, in order to generate a broad distribution of crystallizable chain segments. This condition is typically fulfilled in systems such as random ethylene/α-olefin copolymers, crosslinked polyethylenes, or polymers containing tacticity defects that interrupt otherwise crystallizable sequences [22,23,24,25,26,27].

On the contrary, UHMWPEs used in the manufacture of orthopedic prostheses are homopolymers characterized by long, substantially linear chains, with minimal defects capable of disrupting the linear sequence or enabling thermal fractionation. This is likely one of the reasons why no studies about the application of SSA to medical-grade UHMWPEs are currently available in the literature.

However, we hypothesized that SSA could represent a valuable additional tool for the characterization and discrimination of UHMWPE formulations subjected to different treatments. In particular, while we acknowledged that the thermal fractionation of virgin UHMWPEs may be challenging, we expected that of γ-irradiated UHMWPEs to be more effective due to their crosslinked molecular structure. The formation of tertiary carbons during cross-linking reactions can, in fact, be considered analogous to the structural heterogeneity observed in branched polyethylenes, as both processes result in the disruption of linear crystallizable sequences. Furthermore, we aimed to investigate whether different post-irradiation thermal treatments lead to distinct fractionation behaviors, and whether oxidation or, conversely, the presence of small amounts of stabilizer (α-tocopherol) may influence the outcome. Overall, by proposing SSA as a complementary method of analysis, we highlight its potential not only for advancing the scientific understanding of UHMWPE microstructure but also for providing a practical tool to differentiate commercially relevant formulations.

## 2. Materials and Methods

### 2.1. Materials

Seven UHMWPE GUR1050 (Celanese, Bishop, TX, USA) materials were examined in this study: (1) ram-extruded, as received (UH); (2) in powder form (p-UH); (3) ram-extruded, 30 kGy γ—irradiated in nitrogen (UH30); (4) ram extruded, highly cross-linked with 100 kGy of γ—radiation and remelted at 150 °C for 2 h, then slow cooled to ambient temperature over a period of 24 h (Rem-HXLPE); (5) ram extruded, highly cross-linked with 100 kGy of γ—radiation and annealed at 110 °C for 2 h, then slow cooled to ambient temperature over a period of 24 h (Ann-HXLPE); (6) as (5), shelf-aged 10y at RT, in air (OxAnn-HXLPE); and (7) blended with 0.5% *w*/*w* of α-tocopherol, ram-extruded (VEUH). All samples were obtained from 60 mm diameter, ram-extruded rods, unless otherwise specified.

### 2.2. Micro–FTIR Characterization

All samples were analyzed with an FTIR Microscope (Spectrum Spotlight 300, Perkin Elmer, Shelton, CT, USA) to evaluate the possible presence of oxidation in the material. The ram-extruded rods were microtomed with a POLYCUT S microtome (Reichert-Jung, Wetzlar, Germany) into 170–180 μm thick slices, and two line scans were performed for each sample: one parallel to the microtome’s cutting direction and one in the transverse direction. Line scans were acquired using an acquisition range of 4000–750 cm^−1^, with eight scans/point and a sampling frequency of one point every 200 μm. Oxidation levels were quantified by determining the Oxidation Index (O.I.), which is the ratio of the C=O band area at 1720 cm^−1^ (A_1720_) to the standard methylene band area at 1370 cm^−1^ (A_1370_) [28]:

(1) $$O.\;I.\;=\;\frac{A_{1720}}{A_{1370}}$$

### 2.3. Gel Fraction and Crosslinking Density Measurements

The gel fraction of the irradiated samples was measured according to the procedure outlined in ASTM D-2765 [29]. Round UHMWPE specimens, with a diameter of 5 mm and a mass of approx. 3.5 mg were immersed in boiling p-xylene (Merck Life Science, Milan, Italy) at 140 °C for 8 h to extract the soluble fraction. After extraction, all samples were allowed to dry at room temperature to a constant weight. The gel fraction was calculated using the following equation:

(2) $$Gel\;Fraction=1-\left(\frac{W_{d}}{W_{s}}\times 100\right)$$

where W_s_ is the weight of the original specimen and W_d_ is the weight of the dried gel.

For samples whose gel fraction was ≥98%, the crosslink density was measured by swelling the specimens (diameter: 3 mm; mass: approximately 15 mg) in 25 mL of p-xylene at 130 °C for 2 h; then, the specimens were blot-dried and weighed and the following equation was applied to calculate the crosslink density [30]:

(3) $$\nu_{d}=\;-\frac{ln\;\left(1-q_{s}^{-1}\right)\;+q_{s}^{-1}+\chi_{1}q_{s}^{-2}}{\varphi_{1}(q_{s}^{-\frac{1}{3}}-0.5q_{s}^{-1})}$$

where q_s_ is the polymer’s swelling ratio, Χ_1_ is the Flory interaction parameter (0.33 + 0.55q_s_ for polyethylene–xylene), and φ_1_ is the molar volume of the solvent (136 cm^3^/mol).

### 2.4. Differential Scanning Calorimetry (DSC)

Differential scanning calorimetry was performed using a DSC Discovery Series 250 (TA Instruments, New Castle, DE, USA) equipped with a refrigerating cooling system RCS 40 (TA Instruments). Round specimens with a diameter of 5 mm and a mass of approximately 3.5 mg were tested. The sample in the form of powder was analyzed as received.

#### 2.4.1. Standard Thermal Analysis

A “standard” thermal run was performed on all samples, in order to identify the one with the highest melting point, to be subjected to the Self—Nucleation thermal protocol [31]. The standard thermal protocol employed was: (1) heating ramp at 20 °C/min from 40 °C to 170 °C to erase the sample’s thermal history; (2) holding the samples at 170 °C for 2 min; (3) cooling ramp at 20 °C/min from 170 °C to 40 °C; (4) holding the samples at 40 °C for 2 min; (5) final heating ramp at 20 °C/min from 40 °C to 170 °C. Percent crystallinity was calculated by normalizing the heat of fusion of the sample to that of 100% crystalline polyethylene (293 J/g).

#### 2.4.2. Successive Self–Nucleation and Annealing (SSA) Thermal Protocol

Variables such as the choice of the self-nucleation temperature (T_s_), the time spent at T_s_ (isothermal fractionation time), the temperature interval between two consecutive T_s_ (fractionation window), the scanning rate and the sample mass are known to significantly influence the outcome of an SSA protocol [31]. Accordingly, a series of preliminary experiments was carried out to design a reliable experimental procedure.

In particular, prior to defining the SSA thermal protocol, a Self-Nucleation (SN) analysis was conducted to determine the “Ideal Self-Nucleation Temperature” (T_s,ideal_). This temperature represents the lowest point at which maximum self-nucleation occurs without annealing of the self-nuclei [19]. Since we were comparing a series of similar samples, we chose to use the highest T_s,ideal_ as a constant initial T_s_ for all samples originating from the same ram-extruded lot. The highest T_s,ideal_ (139.5 °C) was determined for the remelted UHMWPE sample (the one with the highest melting point among the ram-extruded series). A separate Self-Nucleation analysis was performed on the sample in the form of powder (p-UH) and a T_s,ideal_ of 133.0 °C was determined.

Concerning the residence time at T_s_ and the fractionation window, different times (5, 7.5, and 10 min) and fractionation intervals (5, 8, and 10 °C) were investigated, and 5 min and 5 °C, respectively, were identified as the optimal parameters. These findings are consistent with the work of Müller et al. [31], who reported that such conditions are generally suitable for most applications.

A high scanning rate is desirable, as it reduces the overall duration of the experiment. However, an increase in the heating rate must be compensated by a reduction in sample mass, in order to avoid superheating effects, as reported by Lorenzo et al. [32]. Accordingly, different scanning rates (10, 20, and 30 °C/min) were investigated in combination with progressively reduced sample masses (9.5, 6.5, and 3.5 mg, respectively). A scanning rate of 20 °C/min and a sample mass of approximately 3.5 mg were identified as the best compromise between experimental time and resolution.

Preliminary analyses were performed in duplicate to assess measurement reproducibility, with variations in the measured melting temperatures remaining within a few tenths of a degree Celsius.

**Figure 1:**
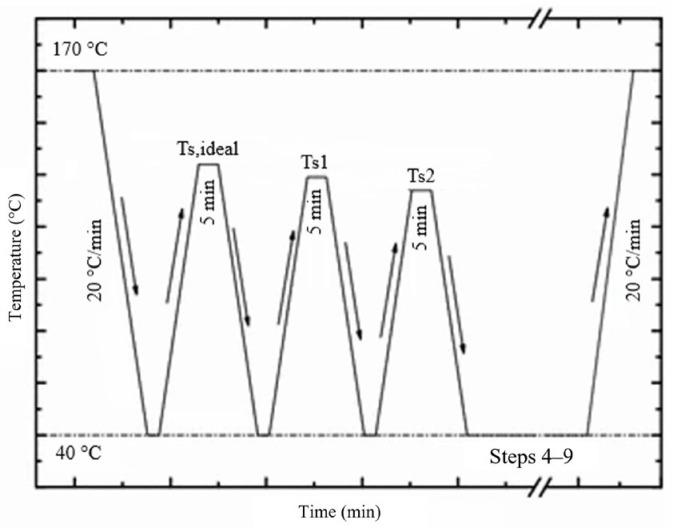
Schematic representation of the SSA thermal protocol employed to perform the thermal fractionation.

The SSA protocol consisted of the following steps (Figure 1) [4,6]:

(a) The sample is held for 3 min at 170 °C (T_MAX_) to erase the crystalline memory.
(b) The sample is cooled at a constant rate of 20 °C/min from 170 °C to 40 °C (T_MIN_) to allow the complete crystallization of the material. The peak crystallization temperature recorded during this cooling ramp is the “standard” crystallization temperature (T_c_).
(c) The sample is heated at 20 °C/min from 40 °C to the T_s,ideal_ measured in the previous Self-Nucleation experiment.
(d) The sample is maintained at T_s_ for 5 min. This isothermal step results in partial melting and, depending on the T_s_, in the annealing of unmelted crystalline fractions.
(e) The sample is cooled at 20 °C/min from T_s,ideal_ to 40 °C, thus the fraction that melted at T_s_ will crystallize. According to the definition of ‘Domains’ by Mueller et al. [4], if the sample lies within Domain I (complete melting domain), crystallization takes place at the standard T_c_, whereas in Domain II (exclusive self-nucleation domain), it occurs at higher temperatures due to self-nucleation. Finally, if the sample lies within Domain III (self-nucleation and annealing domain), crystallization occurs immediately upon cooling, just below T_s_.
(f) Steps (c), (d), and (e) are repeated iteratively, gradually decreasing T_s_ by 5 °C in each sequence. This ΔT corresponds to the fractionation window, which defines the width of the thermal fraction and is kept constant throughout the SSA experiment. The number of repetitions is defined by choosing the value of the fractionation window (5 °C) and fractionation range (40 °C). In this work, the SSA thermal protocol consists of 9 cycles.
(g) The last step involves a heating ramp, at a constant rate of 20 °C/min, to 170 °C. During this ramp, the fractionation profile is recorded.

### 2.5. Deconvolution Procedure

To enable quantitative comparisons of SSA results, melting peaks in the fractionation profiles were separated to quantify their height and area [22]. The deconvolution of the final DSC heating scan into elementary curves was performed using the commercial software OriginPro 8.5, applying the following Gaussian equation:

(4) $$y=\;y_{0}+\;\frac{Ae^{\frac{-4ln\;\left(2\right)+(x-x_{c}{)}^{2}\;}{w^{2}}}}{w\times \sqrt{\frac{\pi }{4ln(2)}}}$$

where y is the heat flow, x is the DSC temperature, y_0_ is the baseline value, A is the peak area, and w is the peak amplitude.

For each thermogram, the baseline was defined from the high-temperature region beyond the melting transitions, where no thermal events were observed, and was anchored prior to fitting. Initial peak positions were assigned based on the experimental maxima of the DSC curves. In the case of overlapping peaks, the experimental signal was resolved into multiple Gaussian components to account for partially superimposed melting events. Peak deconvolution was performed by nonlinear least-squares fitting, and the reliability of the fitting procedure was assessed using the adjusted coefficient of determination (adjusted R^2^), which exceeded 0.983 in all cases, confirming the robustness of the quantitative deconvolution.

## 3. Results

**Figure 2:**
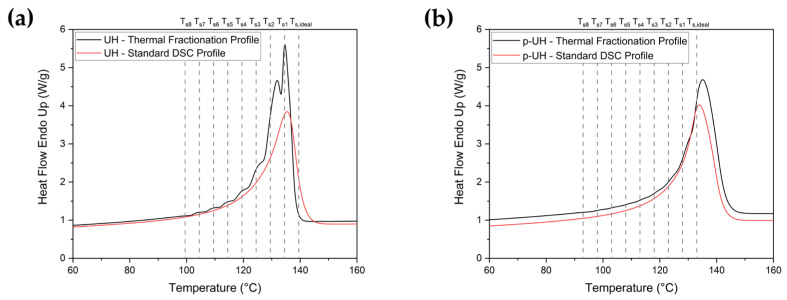
Thermal fractionation profiles and standard DSC profiles of UH (**a**) and p-UH (**b**) samples.

Figure 2a shows the standard DSC run of the as-received, ram-extruded UH along with its thermal fractionation profile obtained by SSA. The thermal properties recorded from the standard DSC runs and from the thermal fractionation experiments are shown in Table 1.

The fractionation profile shows two dominant melting peaks and five low-temperature shoulders. According to the SSA thermal protocol developed in this study, nine T_s_ temperatures were investigated. The first T_s_, 139.5 °C, is the T_s,ideal_, located in Domain II [31]. In this domain, the polymer only self-nucleates without undergoing annealing. Consequently, if nine different values of T_s_ were investigated and only one fell within Domain II, the final fractionation profiles should exhibit eight different melting peaks [31]. In contrast, the fractionation profile of virgin GUR1050 exhibits only seven melting peaks, since the thermal protocol was developed based on a self-nucleation experiment performed on Rem-HXLPE, which displayed a higher melting temperature. Consequently, at the second investigated T_s_ (134.5 °C), the virgin sample still falls within Domain II, corresponding to the exclusive self-nucleation region, whereas it enters Domain III, associated with self-nucleation and annealing, starting from the third investigated T_s_ (129.5 °C).

The relatively evident fractionation of UHMWPE was quite unexpected. Rojas de Gascue et al. [22] reported that HDPE specimens obtained from compression-molded sheets could not be fractionated, since HDPE is a substantially linear polymer in which only minimal interruptions occur along the crystallizable sequences of the chains, making it insensitive to thermal fractionation.

In fact, when SSA is performed on UHMWPE in powder form (p-UH), practically no fractionation occurs and the final heating scan displays a single melting peak (Figure 2b). This observation is consistent with the low level of chain entanglement typically expected in nascent powder, suggesting that the thermal fractionation observed in ram-extruded UHMWPE originates from the entanglements introduced between polymer chains during processing, which act as physical interruptions of the otherwise highly linear methylene sequences. Indeed, during melt processing, UHMWPE is known to undergo the so-called “chain explosion” phenomenon, whose extent increases with molecular weight. This phenomenon strongly enhances the diffusion of chain segments, thereby promoting the formation of an extensively entangled network [33]. Wang et al. demonstrated that careful adjustment of sintering temperature and heating rate can significantly influence the entanglement density of UHMWPE molecular chains [34]. However, since the exact processing parameters used for the consolidation of commercial GUR 1050 are proprietary, no direct information is available to support a more quantitative discussion of the resulting entanglement density.

**Figure 3:**
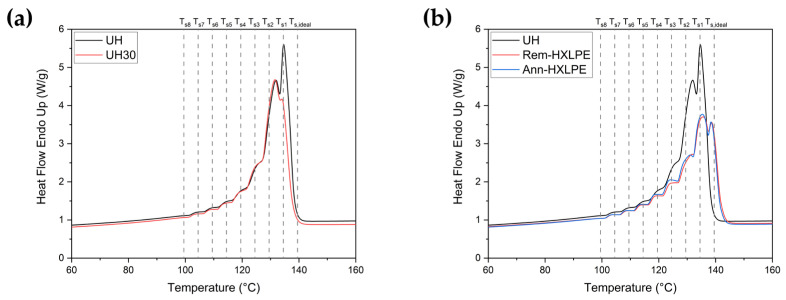
Thermal fractionation profiles comparison of (**a**) UH vs. UH30; (**b**) UH vs. Rem-HXLPE vs. Ann-HXLPE.

Figure 3a compares the final heating scans obtained after applying the SSA protocol to UH and UH30, respectively. Irradiation of UHMWPE with 30 kGy of gamma radiation resulted in a slight decrease in crystallinity (see Table 1). This is a well-known consequence of cross-linking induced by irradiation, which causes interruption of the linear crystallizable methylene sequences, thereby reducing the overall crystallinity. Thermal fractionation reveals that the reduction predominantly involves the highest-melting fraction, associated with the thicker lamellae, whose formation appears to be hindered by the constraints imposed by cross-links.

A different behavior is observed for samples Rem-HXLPE and Ann-HXLPE (Figure 3b). Both samples were irradiated to 100 kGy, but, unlike UH30, they had also been subjected to a thermal treatment after irradiation. As already mentioned, such thermal treatments are commonly performed in commercial biomedical UHMWPE with the scope to induce recombination of the free radicals generated by irradiation. Exposure of UHMWPE to 100 kGy irradiation, followed by thermal treatment, promotes extensive cross-linking, as indicated by the gel fraction values reported in Table 2.

The specific thermal treatment (remelting at 150 °C or annealing at 110 °C) does not appear to significantly influence either the thermal properties or the thermal fractionation behavior. Once again, the overall crystallinity is lower than that of the pristine sample because of the effect of crosslinking (Table 1). Moreover, regardless of the thermal treatment, both samples exhibit crosslink densities within a comparable range (0.33 ± 0.01 and 0.22 ± 0.02 mol/dm^3^ for Rem-HXLPE and Ann-HXLPE, respectively) and very similar thermal fractionation profiles, characterized by eight discrete fractions, as expected for the nine investigated T_s_ values, with the highest-melting fraction appearing just above 138 °C. The appearance of this higher-melting fraction was somewhat unexpected. Following irradiation at 100 kGy and the consequent crosslinking, one would rather expect a reduction in the highest-melting fraction observed in the virgin material, due to the constraints imposed by the crosslinked network, as indeed observed for the UH30 sample. However, unlike UH30, both Rem-HXLPE and Ann-HXLPE underwent an additional thermal treatment after irradiation, and we hypothesize that this step plays a key role in the observed behavior. In particular, we propose that the higher-melting fraction originates from molecular rearrangements induced by prolonged high-temperature exposure after irradiation, which may confine uncrosslinked chains, or segments thereof, containing long methylene sequences, ultimately leading to the formation of a minor population of thicker lamellae.

**Figure 4:**
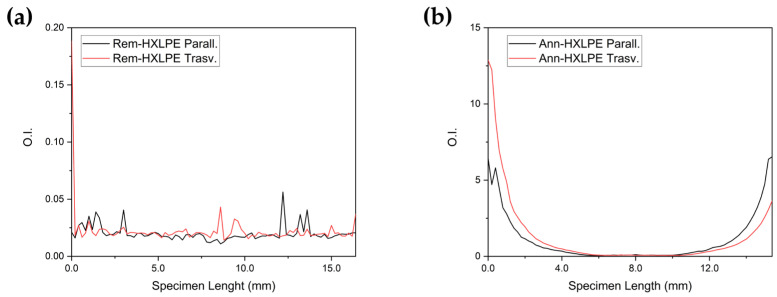
Through-thickness oxidation profiles obtained by line scans in parallel and transverse directions for Rem-HXLPE (**a**) and Ann-HXLPE (**b**). Note the different y-axis scales.

In contrast, the selected thermal treatment has been shown to strongly affect the oxidative stability of the material. Long-term aging resulted in significant differences between the two samples, as illustrated in Figure 4 by their oxidation profiles, obtained from an FTIR line scan across the full thickness of the specimen block. Ann-HXLPE exhibits a complex OI profile, with maxima located close to the sample surface. It is well established in the literature [35] that this phenomenon arises because annealing below the melting temperature leaves a measurable amount of free radicals within the polymer matrix. These residual radicals can subsequently react with oxygen over time, thereby initiating the oxidation cascade. The observed profile is consistent with the different oxygen availability in the surface and inner layers of the material: the highest level of oxidation is found at the surface of the irradiated block, whereas the innermost region, where oxygen availability is limited, remains relatively unaffected.

**Figure 5:**
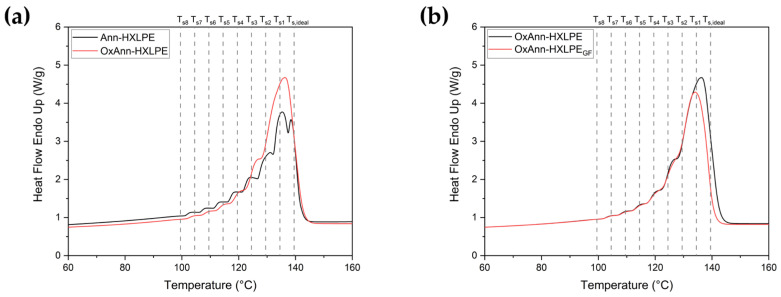
Thermal fractionation profiles comparison: Ann-HXLPE vs. OxAnn-HXLPE (**a**); OxAnn-HXLPE vs. OxAnn-HXLPE_GF_ (**b**).

With the aim of investigating the effect of extensive oxidation on thermal fractionation, we subjected the oxidized surface layer of Ann-HXLPE to the same SSA protocol. The result is shown in Figure 5a.

The overall crystallinity of the OxAnn-HXLPE sample is approximately 11% higher than that of its unoxidized counterpart (Table 1). Furthermore, the thermal fractionation profile shows only six of the original eight fractions, with a predominant single broad peak centered at about 136 °C.

Oxidative degradation is well known to affect polymer crystallization [36]. Mueller and coworkers have investigated the thermal fractionation of pro-oxidant-containing LDPE and LLDPE subjected to environmental or accelerated oxidative degradation [37]. After short-time aging, they observed the development of a new population of thick, high-melting lamellae, which was attributed to a decrease in the molecular mass of the polymer and in chain entanglement, caused by a moderate oxidation level. This phenomenon enhances chain mobility, thereby facilitating crystallization of previously constrained chain segments. On the other hand, when oxidation progresses further, extensive chain scissions limit the average methylene sequence length available to form thick lamellae, resulting in the disappearance of the higher melting fractions and broadening of the lower melting peak.

This behavior is consistent with our observations for OxAnn-HXLPE, where severe oxidation likely induced extensive chain scission, together with the formation of additional irregularities in the form of oxidized groups randomly distributed along the polymer chains. While random chain scission decreases both molecular weight and chain entanglement, thereby enhancing chain mobility and crystallizability, it also generates a highly heterogeneous distribution of chain lengths and crystallizable segments. This, in turn, leads to an equally heterogeneous distribution of lamellar thicknesses, ultimately hindering an effective separation by thermal fractionation.

Further evidence of significant chain scission is provided by the marked reduction in gel fraction observed for this material compared to Ann-HXLPE (Table 2). Figure 5b compares the final heating scans after SSA fractionation for OxAnn-HXLPE and for the gel fraction extracted from the same material (OxAnn-HXLPE_GF_). The comparison clearly indicates that the soluble fraction, consisting of shorter, more degraded, but also more mobile polymer chains, is responsible for the high-temperature melting tail of the broad endothermic peak.

To allow for quantitative comparison of the SSA results, the individual melting peaks were separated and their heights and areas quantified, thus providing the relative contribution of each thermal fraction.

**Figure 6:**
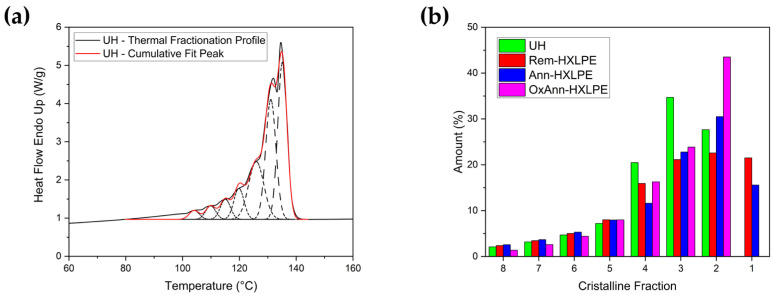
(**a**) Results of the deconvolution analysis performed on the UH sample; (**b**) relative amount (%) of the crystalline fractions generated during SSA in UH, Rem-HXLPE, Ann-HXLPE, and OxAnn-HXLPE, respectively.

Figure 6a presents, as an example, the result of the deconvolution procedure applied to virgin UHMWPE, while Figure 6b summarizes the relative amount, expressed as a percentage, of each crystalline fraction developed during SSA by UH, Rem-HXLPE, Ann-HXLPE, and OxAnn-HXLPE, respectively.

More than 80% of the crystalline fraction of virgin UH is composed of three populations of crystals, melting at approximately 135, 132, and 126 °C (fractions 2, 3, and 4). As already mentioned, because UHMWPE is an almost linear polyethylene with minimal chain defects, its fractionation is difficult and likely based more on chain entanglements than on actual interruptions of the methylene sequence length. On the other hand, after 100 kGy irradiation, followed by either annealing or remelting, a significant crystalline fraction (>15%) is composed of a new, high melting population, whose origin was discussed above. Rem-HXLPE and Ann-HXLPE share a very similar fractionation, because of their similar history; only, Rem-HXLPE exhibits a slightly higher amount of the highest melting fraction 1, likely due to the higher temperature used for the post-irradiation treatment, which allowed for a more efficient chain mobility. The formation of crystalline fraction number 1 occurs mainly at the expense of crystalline fractions 3 and 4, whose amount is reduced compared to that of the virgin sample. When oxidation occurs (OxAnn-HXLPE), the highest melting fraction number 1 disappears, while the amount of the lower melting fractions 4, 3, and particularly 2 increases as a consequence of the enhanced mobility of the oxidatively degraded chains.

Finally, we sought to assess whether the incorporation of small amounts of an antioxidant additive could affect the fractionation behaviour, i.e., whether SSA can be used to characterize antioxidant-containing materials. α-Tocopherol (synthetic vitamin E) is now widely used as an antioxidant in biomedical UHMWPE. Various formulations can be found in commercial products on the market, and the threshold minimum effective amount, as well as the efficacy of different incorporation methods, have been much discussed in the literature [13,14,38]. Since the amount of incorporated additive is generally in the order of less than 0.5 wt%, the a posteriori determination of the actual amount is difficult to obtain with the most common analytical techniques.

**Figure 7:**
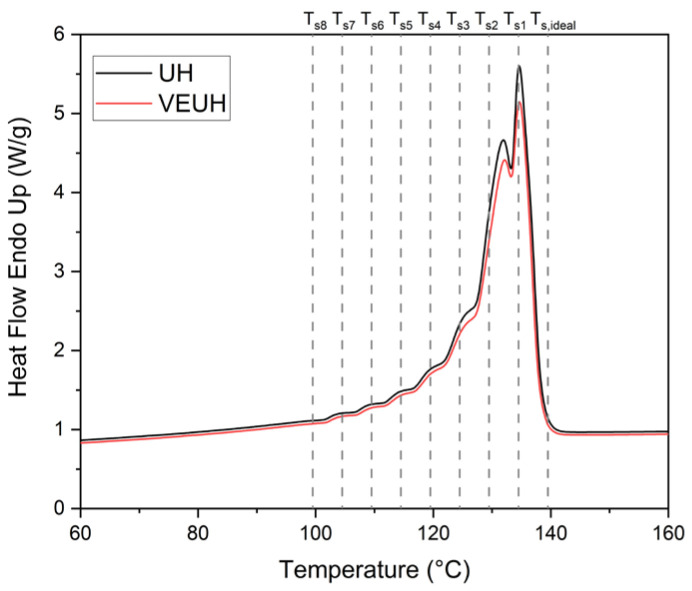
Thermal fractionation profiles of UH and VEUH samples.

Figure 7 shows the thermal fractionation profile of a sample containing 0.5% *w*/*w* of α-tocopherol, compared to that of standard UHMWPE. Although the overall crystallinity of VEUH is slightly lower than that of virgin UH, the two fractionation profiles barely showed any difference, indicating that, unfortunately, SSA is unable to discriminate among virgin and additive-containing materials. This likely occurs because the α-tocopherol concentration, although sufficient to provide oxidative stabilization, is too low to significantly influence lamellar segregation or induce confinement effects capable of measurably affecting crystallization and thermal fractionation behavior. Future investigations involving higher antioxidant concentrations or alternative stabilization strategies could help to further clarify the relationship between antioxidant content, crystallization behavior, and thermal fractionation.

Despite this limitation, the overall results of the study highlight the unique capability of SSA to resolve the distribution of crystalline populations and lamellar thickness heterogeneity within UHMWPE, providing information that cannot be directly obtained from conventional techniques such as standard DSC or FTIR and approaching the level of structural insight typically accessible only through more sophisticated and experimentally demanding techniques such as TEM or SAXS. Similar observations have already been reported in the literature [22], further supporting the potential of SSA as a valuable tool for the investigation of polymer microstructure.

## 4. Conclusions

Thermal fractionation of extruded, medical grade, ultra-high-molecular-weight polyethylene (UHMWPE) can be achieved using the SSA technique. Although medical-grade UHMWPE in powder form is not suitable for fractionation, due to its highly linear structure and the absence of structural irregularities, the extrusion process induces a densely entangled network within the polymer, thereby facilitating molecular segregation.

High-energy irradiation followed by thermal treatments reduces the overall crystallization ability of the polymer, as chain mobility is restricted by the presence of chemical crosslinks. However, the formation of tertiary carbon atoms during irradiation-induced crosslinking disrupts linear crystallizable sequences, while subsequent thermal treatments significantly alter the polymer morphology, leading to the formation of an additional population of high-melting crystallites, as revealed by SSA. In contrast, oxidative degradation reduces molecular weight and enhances chain mobility, resulting in increased crystallinity, albeit at the expense of the ability to undergo fractionation. On the other hand, low concentrations of α-tocopherol used as oxidation stabilizers were found to be insufficient to significantly affect fractionation.

Overall, SSA has proven capable of providing valuable insights into the morphology of biomedical UHMWPE and enabling effective discrimination among commercially available formulations.

## Figures and Tables

**Table 1:** Standard thermal properties of the UHMWPE samples investigated and melting peak fractions obtained after applying the SSA thermal protocol.

	Std. DSC		SSA							
Samples	T_m_ (°C)	Χ_c_ (%)	T_m8_ (°C)	T_m7_ (°C)	T_m6_ (°C)	T_m5_ (°C)	T_m4_ (°C)	T_m3_ (°C)	T_m2_ (°C)	T_m1_ (°C)
p-UH	133.9	48	-	-	-	-	-	-	135.2	-
UH	135.4	47	104.1	109.9	115.1	119.8	125.9	131.8	134.7	-
UH30	133.5	45	103.6	109.1	114.6	119.7	125.2	131.5	134.1	-
Ann-HXLPE	136.5	43	103.7	109.1	114.3	119.4	124.2	131.1	135.3	138.4
Rem-HXLPE	138.6	44	103.9	109.3	114.2	119.3	125.4	131.5	135.5	138.5
OxAnn-HXLPE	130.8	54	104.5	110.1	115.1	121.0	126.9	*	136.4	-
VEUH	132.3	46	104.6	109.4	114.7	119.9	125.2	131.9	134.6	-

* The melting peak corresponding to the third thermal fraction (T_m3_) was not observed because it overlapped with T_m2_.

**Table 2:** Gel fraction of pristine, irradiated, and irradiated/thermally treated UHMWPE samples. Values are reported as mean ± standard deviation (*n* = 3).

Sample	Gel Fraction (%)
UH	-
UH30	96.9 ± 0.8
Rem-HXLPE	98.9 ± 0.1
Ann-HXLPE	98.4 ± 0.2
OxAnn-HXLPE	84.4 ± 4.1

## Data Availability

The original contributions presented in this study are included in the article. Further inquiries can be directed to the corresponding author.
